# The Model for End-stage Liver Disease (MELD) as a predictor of short-term mortality in *Staphylococcus aureus* bloodstream infection: A single-centre observational study

**DOI:** 10.1371/journal.pone.0175669

**Published:** 2017-04-17

**Authors:** Jan A. Roth, Andreas F. Widmer, Sarah Tschudin-Sutter, Marc Dangel, Reno Frei, Manuel Battegay, Balthasar L. Hug

**Affiliations:** 1 Division of Infectious Diseases and Hospital Epidemiology, University Hospital Basel, Basel, Switzerland; 2 University of Basel, Basel, Switzerland; 3 Division of Clinical Microbiology, University Hospital Basel, Basel, Switzerland; 4 Department of Internal Medicine, Kantonsspital Luzern, Lucerne, Switzerland; Amphia Ziekenhuis, NETHERLANDS

## Abstract

**Background:**

Automated laboratory-based prediction models may support clinical decisions in *Staphylococcus aureus* bloodstream infections (BSIs), which carry a particularly high mortality. Small studies indicated that the laboratory-based Model for End-stage Liver Disease (MELD) score is a risk factor for mortality in critically ill patients with infections. For *S*. *aureus* BSIs, we therefore aimed to assess a potential association of the MELD score with mortality.

**Methods:**

In this single-centre observational study, all consecutive patients with a first episode of methicillin-susceptible *S*. *aureus* BSI occurring between 2001 and 2013 were eligible. Relevant patient data were retrieved from our prospective in-house BSI database. We assessed the association of the MELD score at day of BSI onset (range ± two days) with 30-day all-cause mortality using uni- and multivariable logistic regression analysis.

**Results:**

561 patients were included in the final analysis. The MELD score at BSI onset was associated with 30-day mortality in *S*. *aureus* BSIs (odds ratio per 1-point increase, 1.06; 95% confidence interval, 1.03‒1.09; *P* < 0.001). After adjustment for relevant patient and infection characteristics, an increased MELD score remained a predictor of 30-day mortality (adjusted odds ratio per 1-point increase, 1.05; 95% confidence interval, 1.01‒1.08; *P* = 0.005).

**Conclusions:**

In our study population, the MELD score at BSI onset was an independent predictor of mortality in *S*. *aureus* BSIs. We therefore suggest to prospectively validate the MELD score as part of clinical decision support systems in inpatients with suspected or confirmed BSI.

## Introduction

*Staphylococcus aureus* is a leading cause of bloodstream infection (BSI) carrying a high mortality, even if treated with adequate antimicrobial and supportive measures [1, 2]. The considerable mortality stresses the importance of prediction models to support clinical decisions in *S*. *aureus* BSIs.

The Model for End-stage Liver Disease (MELD) is a widely-used risk model, which was initially created to predict mortality in patients with portal hypertension undergoing placement of transjugular intrahepatic portosystemic shunts [3]. Subsequently, it was thoroughly validated as a predictor of mortality among different patient populations across a broad spectrum of liver diseases—primarily to allocate organs for liver transplantation [4–6]. The MELD score incorporates three routine laboratory parameters, i.e. serum creatinine, serum bilirubin, and the International Normalized Ratio (INR), which could enable rapid automated clinical decision support without depending on complex clinical variables.

Small studies indicated that the MELD score is a predictor of mortality in various patient populations with infections or infectious complications [7–9]. We therefore hypothesized that the MELD score is an independent predictor of mortality in *S*. *aureus* BSIs. The overall goal of our study was achieved in terms of establishing that the automatically calculated MELD score might be an independent predictor of mortality in patients with BSIs caused by *S*. *aureus*.

## Methods

### Study design and setting

This was an observational study performed at the University Hospital Basel, an 800-bed tertiary referral centre in Northwestern Switzerland with >35,000 hospitalisations per year; treatment modalities cover all surgical and medical specialties including kidney and bone marrow transplantations. The Ethics Committee of Northwestern and Central Switzerland (EKNZ) approved this study with a waiver on written/oral informed consent (study number 2016–01515).

### Patient selection

Patients aged ≥18 years with a first methicillin-susceptible *S*. *aureus* BSI episode occurring between January 2001 and December 2013 were eligible for the study. We excluded recurrent BSI episodes (i), patients with a missing 30-day follow-up (ii), patients with missing MELD parameters at BSI onset (iii), patients with missing medication data on vitamin K antagonists and novel oral anticoagulants (iv), and patients treated at BSI onset with a vitamin K antagonist (v) or a novel oral anticoagulant (vi) because of their effect on the INR.

### Data collection

We extracted relevant data from our prospective in-house BSI database, which includes demographic, microbiological, routine laboratory, treatment and outcome data of all patients with positive blood cultures. The MELD score was retrospectively calculated according to the United Network for Organ Sharing modifications [4, 10] using the first available routine laboratory data set at day of BSI onset (range ± two days).

### Study definitions

An episode of *S*. *aureus* BSI was defined as the detection of *S*. *aureus* in one or more blood cultures with or without additional identification of a contaminant according to CDC recommendations [11]. A recurrent BSI episode was defined as the detection of *S*. *aureus* in a blood culture >7 days after the last identification from blood culture. An episode of BSI without a definite source of infection was defined as BSI of primary origin. The time stamp of the first positive blood culture by the in-house microbiological laboratory was defined as the day of BSI onset. Onset of a BSI after more than two days of hospitalisation was interpreted as hospital-acquired BSI. The diagnosis of liver cirrhosis was collected retrospectively from medical records and verified by reviewing ultrasonographic, laboratory, endoscopic, and pathological reports [12]. Immunosuppression was defined as described previously [13]. In brief, the definition included the presence of conditions leading to immunosuppression (e.g. end-stage renal failure, haematologic malignancies), immunological/inflammatory diseases requiring immunosuppressive therapy, or an absolute neutrophil count <500/μl. An adequate empirical antimicrobial therapy was defined in retrospect as providing coverage against methicillin-susceptible *S*. *aureus*.

### Microbiology

During the entire study period, rapid processing and incubation of the blood cultures were ensured 24 hours per day using the same blood culture system with at least one pair of aerobic/anaerobic bottles (BacT/ALERT; bioMérieux, Durham, North Carolina, Unites States of America).

### Statistical analysis

Patient and infection characteristics were presented as proportions, means with standard deviation and as medians with interquartile ranges (IQR) as appropriate. For 30-day survivors and non-survivors, we assessed unadjusted differences in the patient and infection characteristics with the use of the Chi-square test or Fisher’s exact test for categorical variables and the Student’s t-test or the Wilcoxon test for continuous variables, depending on the data distribution.

We assessed the association of the MELD score at BSI onset with 30-day all-cause mortality by means of uni- and multivariable logistic regression analysis; The multivariable logistic regression model was built using a forward selection procedure (at *P* < 0.10) for the following co-variables: Age (continuous), gender, Charlson Comorbidity Index [14] at BSI onset, days of hospitalisation prior to BSI onset, BSI origin (primary or secondary), and presence or absence of liver cirrhosis, immunosuppression, intravenous drug use, and any surgery in the last 30 days prior to BSI onset [15–17]. The dichotomous origin of BSIs was used in the multivariable model, as *S*. *aureus* BSIs of primary origin have been described as independent predictor of mortality relative to other BSI sources [17].

Subsequently, we stratified the patients into the following MELD categories at BSI onset: scores <10, 10‒15, and/or >15. The association of these MELD categories with 30-day all-cause mortality was analysed by means of a univariable Cox proportional hazards model and comparison of the corresponding Kaplan-Meier curves using the Log-rank test.

For patients with and without liver cirrhosis, we compared laboratory and outcome parameters with the use of the Chi-square test or the Fisher’s exact test for categorical variables and the Student’s t-test or the Wilcoxon test for continuous variables, depending on the data distribution. We analysed all data using SPSS, version 22 (SPSS; IBM, Chicago, Illinois, United States of America); All P-values are two-sided with values ≤0.05 being considered as significant.

## Results

### Patient selection

**Fig 1:**
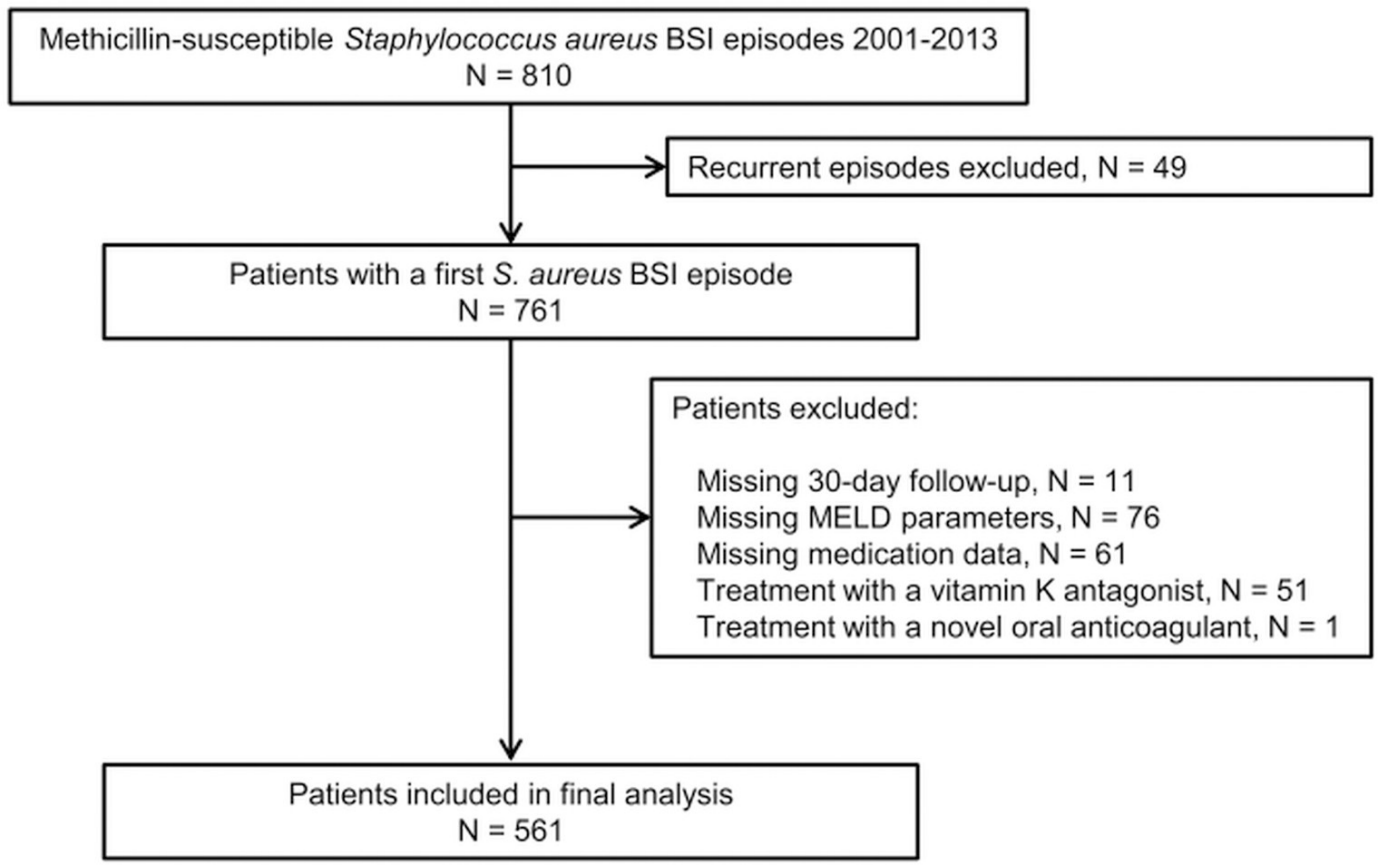
Selection of Patients with *Staphylococcus aureus* Bloodstream Infection for Final Analysis. Abbreviations: BSI, bloodstream infection; MELD, Model for End-stage Liver Disease. Sixty-one patients were excluded due to missing medication data on vitamin K antagonists and novel oral anticoagulants.

Within the 13-year study period, 788 patients had a first *S*. *aureus* BSI episode caused by methicillin-resistant *S*. *aureus* (MRSA; n = 27) or methicillin-susceptible *S*. *aureus* (n = 761). Seven hundred sixty-one patients with a first episode of methicillin-susceptible *S*. *aureus* BSI were eligible for the study (Fig 1). Of these, 561 were included in the final analysis after exclusion of 11 patients with a missing 30-day follow-up, 76 patients with missing MELD parameters at BSI onset, 61 patients with missing medication data, and 52 patients treated with a vitamin K antagonist or a novel oral anticoagulant.

### Demographic, laboratory and infection characteristics

Overall, the median age was 65.5 years (IQR, 49.4‒76.7 years) and 36.2% (203/561) were female (Table 1). At BSI onset, the median MELD score was 9.8 (IQR, 7.5‒16.2) with a corresponding median INR, serum creatinine and bilirubin of 1.1 (IQR, 1.0‒1.3), 89.0 μmol/l (IQR, 66.0‒134.0 μmol/l) and 11.0 μmol/l (IQR, 7.5‒16.2 μmol/l), respectively. At BSI onset (± two days), the median MELD score and the corresponding laboratory values did slightly change (S1 Table).

**Table 1:** Demographic, Laboratory and Infection Characteristics of Patients with a *Staphylococcus aureus* Bloodstream Infection (n = 561).

Variable	All patients n = 561	30-day survivors n = 463	30-day non-survivors n = 98	P-value^a^
― Age [years], median (IQR)	65.5 (49.4‒76.7)	61.4 (46.0‒74.2)	69.7 (61.8‒80.1)	**<0.001**
― Female gender, n (%)	203 (36.2)	164 (35.4)	39 (39.8)	0.413
**MELD score and laboratory parameters at BSI onset,**^b^ **median (IQR)**				
― INR	1.1 (1.0‒1.3)	1.1 (1.0‒1.3)	1.2 (1.0‒1.5)	**0.017**
― Serum creatinine [μmol/l]	89.0 (66.0‒134.0)	86.0 (66.0‒127.0)	115.5 (81.0‒178.5)	**<0.001**
― Serum bilirubin [μmol/l]	11.0 (7.5‒18.5)	11.0 (7.0‒17.0)	13.0 (8.0‒32.0)	**0.003**
― MELD score	9.8 (7.5‒16.2)	9.4 (7.5‒15.4)	13.5 (9.1‒20.8)	**<0.001**
**Comorbidities and risk factors at BSI onset**				
― Charlson Comorbidity Index,^c^ median (IQR)	3 (1‒4)	2 (1‒4)	4 (2‒6)	**<0.001**
― Liver cirrhosis,^d^ n (%)	41 (7.4)	28 (6.1)	13 (13.5)	**0.011**
― Diabetes mellitus type 2, n (%)	124 (22.1)	102 (22.1)	22 (22.4)	0.936
― Any surgery in the last 30 days prior to BSI onset, n (%)	138 (24.6)	123 (26.6)	15 (15.3)	**0.019**
― Intravenous drug use, n (%)	98 (17.5)	91 (19.7)	7 (7.1)	**0.003**
― Immunosuppression,^e^ n (%)	158 (28.2)	127 (27.4)	31 (31.6)	0.401
**BSI and treatment characteristics**				
― Days of hospitalisation prior to BSI onset, median (IQR)	1 (0‒4)	1 (0‒3)	1 (0‒5)	0.064
― Hospital-acquired BSI, n (%)	251 (44.7)	206 (44.5)	45 (45.9)	0.796
― Focus of BSI, n (%)				
―― Intravascular catheters/foreign material	150 (26.7)	128 (27.6)	22 (22.4)	0.590
―― Respiratory tract	45 (8.0)	35 (7.6)	10 (10.2)	
―― Skin and soft tissue	160 (28.5)	134 (28.9)	26 (26.5)	
―― Osteomyelitis/arthritis	44 (7.8)	34 (7.3)	10 (10.2)	
―― Endocarditis	23 (4.1)	17 (3.7)	6 (6.1)	
―― Other/unknown	139 (24.8)	115 (24.8)	24 (24.5)	
― Adequate empirical antimicrobial therapy,^f^ n (%)	521 (94.6)	431 (94.9)	90 (92.8)	0.397

Abbreviations: BSI, bloodstream infection; INR, International Normalized Ratio; IQR, interquartile range; MELD, Model for End-stage Liver Disease.

^a^ Comparison of 30-day survivors and 30-day non-survivors.

^b^ At day of BSI onset (± two days), the first available laboratory value was taken. The MELD was calculated according to the United Network for Organ Sharing modifications [4, 10].

^c^ Missing data for nine patients.

^d^ Diagnoses of liver cirrhosis were retrospectively collected from medical records [12]. The proportion was expressed as valid percentage; missing data for six patients.

^e^ Immunosuppression was defined as stated elsewhere [13].

^f^ Valid percentage; missing data for ten patients.

The patients suffered from various comorbidities with a median Charlson Comorbidity Index at BSI onset of 3 (IQR, 1‒4) (Table 1); 17.5% (98/561) of patients were intravenous drug users and 28.2% (158/561) were immunosuppressed.

In regard to the BSI characteristics, 44.7% of BSIs (251/561) in the study population were hospital-acquired with the most frequent source being skin/soft tissue and intravascular catheters/foreign materials in 28.5% (160/561) and 26.7% (150/561) of patients, respectively. The 30-day and in-hospital all-cause mortality was 17.5% (98/561) and 18.7% (105/561), respectively.

### Model for End-stage Liver Disease: Association with mortality

At BSI onset, the MELD parameters creatinine and bilirubin were positively associated with 30-day all-cause mortality in univariable analysis (odds ratio [OR] per 1 μmol/l increment, 1.002; 95% confidence interval [CI], 1.001‒1.003; *P* = 0.005 and OR, 1.011; 95% CI, 1.005‒1.018; *P* < 0.001), whereas the INR was not significantly associated with 30-day all-cause mortality (*P* = 0.686; S2 Table). In multivariable analysis adjusting for the MELD parameters, creatinine and bilirubin remained positively associated with 30-day all-cause mortality (adjusted OR per 1 μmol/l increment, 1.002; 95% CI, 1.001‒1.003; *P* = 0.006 and adjusted OR, 1.011; 95% CI, 1.005‒1.018; *P* < 0.001). The positive association of creatinine and bilirubin at BSI onset was also observed with in-hospital mortality in uni- and multivariable analysis (S3 Table).

In univariable analysis, the MELD score at BSI onset was positively associated with 30-day all-cause mortality (OR per 1-point increment, 1.06; 95% CI, 1.03‒1.09; *P* < 0.001) (Table 2). After adjustment for relevant patient and infection characteristics, the MELD score at BSI onset remained positively associated with 30-day all-cause mortality (adjusted OR per 1-point increment, 1.05; 95% CI, 1.01‒1.08; *P* = 0.005). The positive association of the MELD score at BSI onset was also observed with in-hospital mortality in uni- and multivariable analysis (S4 Table).

**Table 2:** Predictors of 30-Day All-Cause Mortality in Patients with *Staphylococcus aureus* Bloodstream Infection (n *=* 561); Univariable and Multivariable Analyses.

Variables at BSI onset	Univariable OR (95% CI)	Univariable P-value	Adjusted OR^a^ (95% CI)	Adjusted P-value
Age in years^b^	1.03 (1.02‒1.05)	**<0.001**	1.02 (1.01‒1.04)	**0.004**
Male gender	0.83 (0.53‒1.30)	0.413	—	**—**
MELD score^b^^,^^c^	1.06 (1.03‒1.09)	**<0.001**	1.05 (1.01‒1.08)	**0.005**
Charlson Comorbidity Index^b^	1.24 (1.13‒1.36)	**<0.001**	1.20 (1.08‒1.33)	**0.001**
Liver cirrhosis	2.41 (1.20‒4.85)	**0.014**	—	—
Immunosuppression	1.22 (0.76‒1.96)	0.401	—	—
Intravenous drug use	0.31 (0.14‒0.70)	**0.005**	—	—
Surgery in the last 30 days prior to BSI onset^d^	0.50 (0.28‒0.90)	**0.021**	—	—
Days of hospital stay prior to BSI onset^b^	1.01 (0.98‒1.03)	0.467	—	—
Primary origin of BSI^e^	1.83 (1.10‒3.03)	**0.019**	1.98 (1.16‒3.38)	**0.013**

Abbreviations: BSI, bloodstream infection; CI, confidence interval; MELD, Model for End-stage Liver Disease; OR, odds ratio.

^a^ The multivariable logistic regression model was built using a forward selection procedure at P <0.10 for the listed co-variables.

^b^ Per 1-unit increment.

^c^ First MELD at day of BSI onset ± two days.

^d^ The reference category are patients without a surgery in the last 30 days prior to BSI onset.

^e^ The reference category is ‘BSIs of secondary origin’, i.e. BSIs with a definite source of infection.

**Fig 2:**
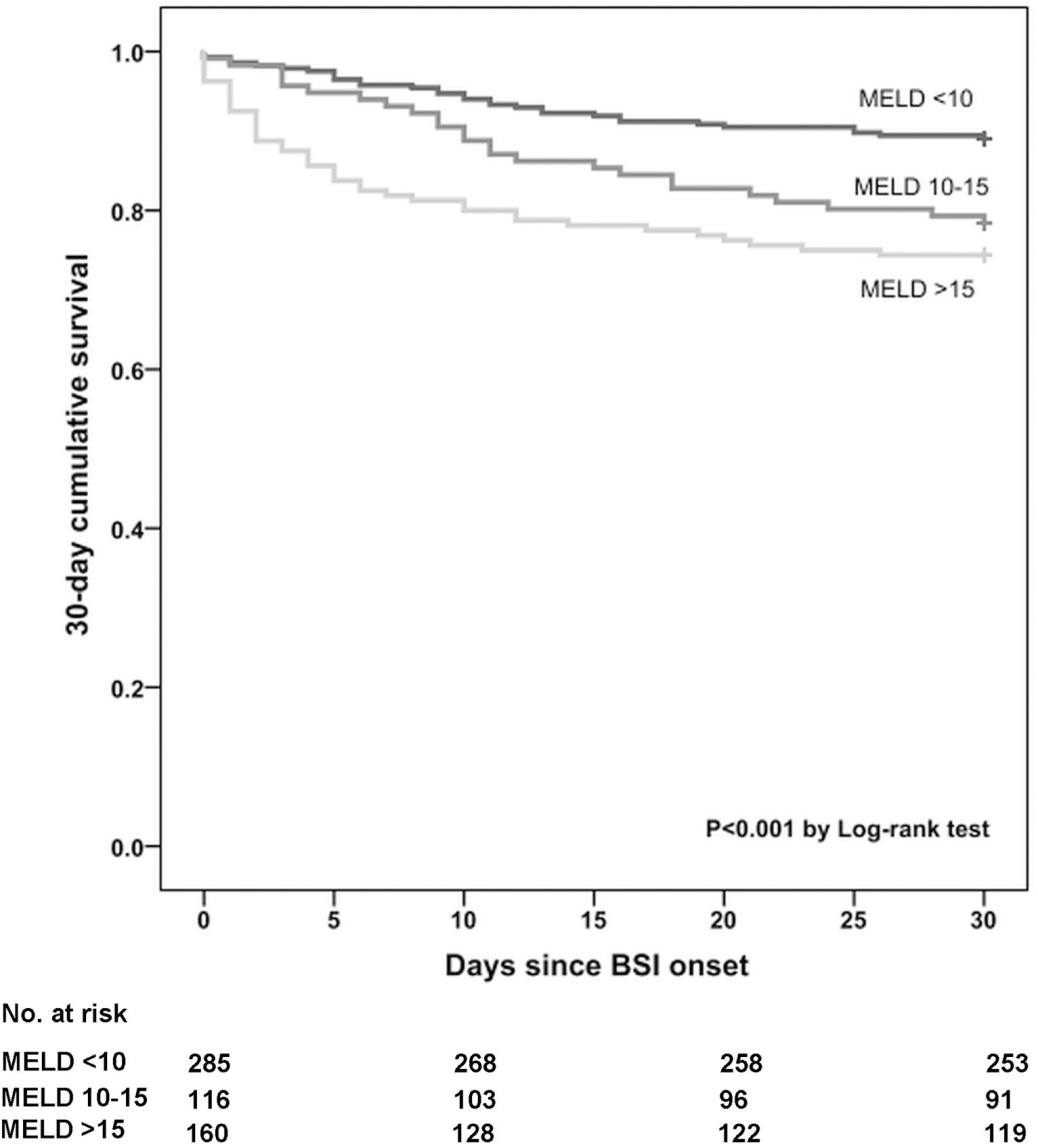
Survival Curves in Patients with *Staphylococcus aureus* Bloodstream Infection and a MELD Score at Infection Onset of <10, 10‒15, or >15 Points; Kaplan-Meier estimates. Abbreviations: BSI, bloodstream infection; MELD, Model for End-stage Liver Disease.

In the univariable Cox proportional hazards model, a MELD score at BSI onset of 10‒15 points and >15 points (reference, <10 points) was significantly associated with an increased hazard ratio (HR) for death before day 30 (HR, 2.06; 95% CI, 1.21‒3.48; *P* = 0.007, and HR, 2.65; 95% CI, 1.66‒4.22; *P* < 0.001) (Table 3); the corresponding Kaplan-Meier survival curves are depicted in Fig 2.

**Table 3:** 30-Day All-Cause Mortality in Patients with *Staphylococcus aureus* Bloodstream Infection Stratified by the MELD Score Category at Bloodstream Infection Onset (n = 561).

MELD score^a^ at BSI onset	Total patients, n (%)	Death within 30 days, *n* (%)	HR^b^ for death within 30 days (95% CI)	P-value^b^
<10 points	285 (50.8)	32 (11.2)	1	—
10‒15 points	116 (20.7)	25 (21.6)	2.06 (1.21‒3.48)	**0.007**
>15 points	160 (28.5)	41 (25.6)	2.65 (1.66‒4.22)	**<0.001**

Abbreviations: BSI, bloodstream infection; CI, confidence interval; HR, hazard ratio; MELD, Model for End-stage Liver Disease.

^a^ First MELD score at day of BSI onset ± two days.

^b^ Survival analysis of the MELD categories (reference: MELD score <10 points) using a univariable Cox proportional hazards model.

Patients with liver cirrhosis (41/555) had a higher MELD score at BSI onset and a higher 30-day all-cause mortality than patients without liver cirrhosis (514/555) (*P* <0.001 and *P* = 0.011, respectively; S5 Table).

## Discussion

In our study population consisting of patients with methicillin-susceptible *S*. *aureus* BSI, the laboratory-based MELD score was an independent predictor of short-term mortality. We focused on a leading pathogen in BSIs and excluded MRSA cases since empirical antimicrobial therapy did not cover MRSA.

The results of this study are in line with several small observational studies indicating that the MELD score might be a predictor of mortality in patients with serious infections—with or without concomitant liver disease [7–9]: In a recent study by Juntermanns et al., liver transplant recipients with a pretransplant MELD of ≥31 points were at higher risk for dying due to septicaemia than individuals with a lower pretransplant MELD score (*P* < 0.05) [9]. In a retrospective study, patients with liver cirrhosis and bacterial infections (in 69.6% of patients [369/530] due to a spontaneous bacterial peritonitis) had an eight percent increase in the mortality rate with every one point rise in the MELD score [8]. Another study demonstrated a good performance of the MELD for mortality prediction in patients with *Vibrio vulnificus* skin and soft tissue infections (n = 39) [7].

If carefully validated and modified to accurately predict mortality in BSIs, the MELD could offer an original opportunity for automated clinical decision support in BSIs without relying on complex clinical variables, which is a major barrier to the application of most mortality prediction scores in daily clinical routine [16, 18, 19]. Regarding clinical decision support, the MELD fulfils important preconditions for a successful prediction model: It incorporates three readily available laboratory variables, which are routinely and repeatedly measured in most inpatients, and which have been demonstrated to be good predictive markers of mortality in patients with and without infections [20–24].

For the observed association of the MELD parameters with mortality, we did not investigate potential underlying pathophysiological mechanisms, as has partly been elucidated for other routine laboratory parameters in BSIs and sepsis (e.g. thrombocyte count) [25]. We speculate that the MELD is a surrogate marker for the presence and severity of a variety of diseases (e.g. kidney injury, liver diseases, malignancies), which may be used for risk stratification and risk adjustment.

In our study, the INR at BSI onset was—in contrast to creatinine and bilirubin—not associated with mortality in uni- and multivariable analysis, although we excluded patients on vitamin K antagonists and novel oral anticoagulants, which could have biased the results. A positive association of the INR with mortality was demonstrated in high-risk patients with sepsis or trauma [26–29]. Conversely, in our study population, only about 30% of patients were admitted to an intensive care unit during the initial hospitalisation period and the INR at BSI onset was rather low with a median of 1.1 (IQR, 1.0‒1.3).

Our study has several limitations. First, we had to exclude 26% of patients which might have led to a selection bias possibly overestimating the effect of the MELD score on mortality, as patients with unmeasured MELD parameters on hospital admission may be healthier than patients, in whom the serum creatinine, bilirubin and INR had been obtained as part of the diagnostic workup. However, only eleven patients had to be excluded due to a missing 30-day follow-up in this hospital-wide observational study. Second, although we have adjusted for important patient and infection characteristics in our analysis, we cannot exclude that unmeasured confounders might have remained, which could have biased our estimates (e.g. time to adequate antimicrobial therapy). Third, our study was performed at a single centre, which may limit the generalizability of our results. In this context, it is important to note that the results of our study do not apply to patients on vitamin K antagonists and novel oral anticoagulants, as these individuals had been excluded from the analysis. Fourth, we did not assess patients with BSIs caused by pathogens other than *S*. *aureus*—particularly microorganisms with less pathogenic properties such as *Escherichia coli*. Fifth, there are studies suggesting an interlaboratory variability in all three components of the MELD score with a mean interlaboratory difference of about five MELD points [30], which may have further reduced the generalizability of our results.

In conclusion, in our study population, the MELD score at BSI onset was an independent predictor of mortality in *S*. *aureus* BSIs. We therefore suggest to prospectively validate the MELD score as part of clinical decision support systems in inpatients with suspected or confirmed BSI.

## Supporting information

S1 Table Dynamics of the Model for End-Stage Liver Disease and the Corresponding Laboratory Parameters at Onset of *Staphylococcus aureus* Bloodstream Infection (± two Days). (DOCX)

S2 Table Predictors of 30-Day All-Cause Mortality in Patients with *Staphylococcus aureus* Bloodstream Infection (n *=* 561); Univariable and Multivariable Analyses of the MELD Parameters at Onset of Bloodstream Infection. (DOCX)

S3 Table Predictors of In-Hospital All-Cause Mortality in Patients with *Staphylococcus aureus* Bloodstream Infection (n = 561); Univariable and Multivariable Analyses of the Model for End-Stage Liver Disease Parameters at Bloodstream Infection Onset. (DOCX)

S4 Table Predictors of In-Hospital All-Cause Mortality in Patients with *Staphylococcus aureus* Bloodstream Infection (n = 561); Univariable and Multivariable Analyses. (DOCX)

S5 Table Comparison of Laboratory Parameters and Mortality in Patients with and without Liver Cirrhosis and Concomitant *Staphylococcus aureus* Bloodstream Infection (n = 555; missing data for six patients). (DOCX)
