# Supplementary material for: The Model for End-stage Liver Disease (MELD) as a predictor of short-term mortality in Staphylococcus aureus bloodstream infection: A single-centre observational study
Source: PLoS One. 2017 Apr 17;12(4):e0175669. doi: 10.1371/journal.pone.0175669 (PMC5393572; doi:10.1371/journal.pone.0175669)
Supplement: S1 Table — (DOCX) [file pone.0175669.s001.docx]

**S1 Table. Dynamics of the Model for End-Stage Liver Disease and the Corresponding Laboratory Parameters at Onset of *Staphylococcus aureus* Bloodstream Infection (± two Days).**

|  | **Day -2** | **Day -1** | **BSI onset^a^**  **(day 0)** | **Day +1** | **Day +2** |
| --- | --- | --- | --- | --- | --- |
|  | **Complete laboratory data set** | | | | |
|  | n = 93 | n = 273 | n = 354 | n = 275 | n = 240 |
| **Parameter,^b^ median (IQR)** |  |  |  |  |  |
| INR | 1.1  (1.0‒1.5) | 1.1  (1.0‒1.5) | 1.1  (1.0‒1.5) | 1.2  (1.1‒1.4) | 1.1  (1.0‒1.4) |
| Serum creatinine [µmol/l] | 84.5  (66.2‒119.7) | 91.0  (67.0‒138.0) | 96.0  (67.0‒150.5) | 92.0  (65.5‒135.5) | 82.5  (62.0‒137.0) |
| Serum bilirubin  [µmol/l] | 10.0  (6.0‒16.0) | 11.5  (8.0‒19.2) | 12.0  (8.0‒20.0) | 11.0  (7.0‒19.0) | 10.0  (7.0‒18.0) |
| MELD score | 8.9  (6.4‒17.6) | 10.1  (7.2‒15.3) | 11.0  (7.6‒18.5) | 11.3  (7.9‒17.8) | 10.6  (7.5‒18.0) |

Abbreviations: BSI, bloodstream infection; INR, International Normalized Ratio; IQR, interquartile range; MELD, Model for End-stage Liver Disease.

N indicates the number of patients.

^a^ Defined as day of the first positive blood culture.

^b^ For the specific laboratory parameters, the first value was taken for each day.
