# Supplementary material for: The Model for End-stage Liver Disease (MELD) as a predictor of short-term mortality in Staphylococcus aureus bloodstream infection: A single-centre observational study
Source: PLoS One. 2017 Apr 17;12(4):e0175669. doi: 10.1371/journal.pone.0175669 (PMC5393572; doi:10.1371/journal.pone.0175669)
Supplement: S3 Table — (DOCX) [file pone.0175669.s003.docx]

**S3 Table. Risk Factors for In-Hospital All-Cause Mortality in Patients with *Staphylococcus aureus* Bloodstream Infection (n = 561); Univariable and Multivariable Analyses of the Model for End-Stage Liver Disease Parameters at Bloodstream Infection Onset.**

| **MELD parameter^a^**  **at BSI onset** | **Univariable OR^b^**  **(95% CI)** | **Univariable**  **P-value** | **Adjusted OR^b^**  **(95% CI)** | **Adjusted**  **P-value** |
| --- | --- | --- | --- | --- |
| INR | 1.050 (0.878‒1.257) | 0.592 | — | — |
| Serum creatinine [µmol/l] | 1.002 (1.001‒1.004) | **<0.001** | 1.002 (1.001‒1.004) | **<0.001** |
| Serum bilirubin [µmol/l] | 1.012 (1.005‒1.018) | **<0.001** | 1.012 (1.005‒1.018) | **<0.001** |

Abbreviations: BSI, bloodstream infection; CI, confidence interval; INR, International Normalized Ratio; MELD, Model for End-stage Liver Disease; OR, odds ratio.

^a^ At the day of BSI onset (± two days), the first available laboratory value was taken.

^b^ Per 1-unit increment (adjusted for the MELD parameters).
