# Supplementary material for: The Model for End-stage Liver Disease (MELD) as a predictor of short-term mortality in Staphylococcus aureus bloodstream infection: A single-centre observational study
Source: PLoS One. 2017 Apr 17;12(4):e0175669. doi: 10.1371/journal.pone.0175669 (PMC5393572; doi:10.1371/journal.pone.0175669)
Supplement: S4 Table — (DOCX) [file pone.0175669.s004.docx]

**S4 Table. Predictors of In-Hospital All-Cause Mortality in Patients with *Staphylococcus aureus* Bloodstream Infection (n = 561); Univariable and Multivariable Analyses.**

| **Variables at BSI onset** | **Univariable OR**  **(95% CI)** | **Univariable**  **P-value** | **Adjusted OR^a^**  **(95% CI)** | **Adjusted**  **P-value** |
| --- | --- | --- | --- | --- |
| Age [years]^b^ | 1.03 (1.02‒1.04) | **<0.001** | 1.02 (1.01‒1.04) | **0.005** |
| Male gender | 0.95 (0.61‒1.47) | 0.821 | — | — |
| MELD score^b,c^ | 1.06 (1.03‒1.09) | **<0.001** | 1.05 (1.02‒1.08) | **0.002** |
| Charlson Comorbidity Index^b^ | 1.27 (1.16‒1.40) | **<0.001** | 1.25 (1.12‒1.38) | **<0.001** |
| Liver cirrhosis | 2.79 (1.42–5.49) | **0.003** | — | — |
| Immunosuppression | 1.28 (0.81‒2.03) | 0.287 | — | — |
| Intravenous drug use | 0.33 (0.16‒0.71) | **0.005** | — | — |
| Surgery in the last 30 days prior to BSI onset^d^ | 0.63 (0.36‒1.07) | 0.088 | — | — |
| Days of hospital stay prior to BSI onset^b^ | 1.02 (1.00‒1.05) | **0.036** | — | — |
| Primary origin of BSI^e^ | 1.90 (1.16‒3.11) | **0.011** | 2.11 (1.24‒3.59) | **0.006** |

Abbreviations: BSI, bloodstream infection; CI, confidence interval; MELD, Model for End-stage Liver Disease; OR, odds ratio.

^a^ The multivariable logistic regression model was built using a forward selection procedure at P <0.10 for the listed co-variables.

^b^ Per 1-unit increment.

^c^ First MELD score at day of BSI onset (± two days).

^d^ The reference category are patients without a surgery in the last 30 days prior to BSI onset.

^e^ The reference category is ‘BSIs of secondary origin’, i.e. BSIs with a definite source of infection.
