# Supplementary material for: The Model for End-stage Liver Disease (MELD) as a predictor of short-term mortality in Staphylococcus aureus bloodstream infection: A single-centre observational study
Source: PLoS One. 2017 Apr 17;12(4):e0175669. doi: 10.1371/journal.pone.0175669 (PMC5393572; doi:10.1371/journal.pone.0175669)
Supplement: S5 Table — (DOCX) [file pone.0175669.s005.docx]

**S5 Table. Comparison of Laboratory Parameters and Mortality in Patients with and without Liver Cirrhosis and Concomitant *Staphylococcus aureus* Bloodstream Infection (n = 555; missing data for six patients).**

| **Variables** | **Liver cirrhosis,**  **n = 41** | **No liver cirrhosis, n = 514** | **P-value** |
| --- | --- | --- | --- |
| **Laboratory parameter at BSI onset,^a^ median (IQR)** |  |  |  |
| — INR | 1.4 (1.2‒1.9) | 1.1 (1.0‒1.3) | **<0.001** |
| — Serum creatinine [µmol/l] | 116.0 (79.0‒175.0) | 87.5 (66.0‒131.0) | **0.039** |
| — Serum bilirubin  [µmol/l] | 44.0 (26.0‒90.0) | 11.0 (7.0‒16.0) | **<0.001** |
| — MELD score | 18.5 (14.0‒21.4) | 9.4 (7.5‒15.0) | **<0.001** |
| **Outcome, n (%)** |  |  |  |
| 30-day all-cause mortality | 13 (31.7) | 83 (16.1) | **0.011** |
| In-hospital all-cause mortality | 15 (36.6) | 88 (17.1) | **0.002** |

Abbreviations: BSI, bloodstream infection; IQR, interquartile range; MELD, Model for End-stage Liver Disease.

^a^ At the day of BSI onset (± two days), the first laboratory value was taken.
